# Can virtual reality improve traditional anatomy education programmes? A mixed-methods study on the use of a 3D skull model

**DOI:** 10.1186/s12909-020-02255-6

**Published:** 2020-10-31

**Authors:** Shi Chen, Jiawei Zhu, Cheng Cheng, Zhouxian Pan, Lingshan Liu, Jianhua Du, Xinhua Shen, Zhen Shen, Huijuan Zhu, Jihai Liu, Hua Yang, Chao Ma, Hui Pan

**Affiliations:** 1 Department of Endocrinology, Endocrine Key Laboratory of Ministry of Health, Peking Union Medical College Hospital (PUMCH), Chinese Academe of Medical Sciences & Peking Union Medical College (CAMS & PUMC), Beijing, 100730 China; 2 National Virtual Simulation Laboratory Education Center of Medical Sciences, PUMCH, CAMS & PUMC, Beijing, 100730 China; 3 Eight-year Program of Clinical Medicine, PUMCH, CAMS & PUMC, Beijing, 100730 China; 4 Department of Human Anatomy, Histology and Embryology, Institute of Basic Medical Sciences, Neuroscience Center, Chinese Academy of Medical Sciences, School of Basic Medicine, Peking Union Medical College, Beijing, 100005 China; 5 The State Key Laboratory of Management and Control for Complex Systems, Institute of Automation, Chinese Automation, Chinese Academy of Sciences (CASIA), Beijing, 100190 China; 6 Department of Emergency, PUMCH, CAMS & PUMC, Beijing, 100730 China; 7 Department of Otolaryngology-Head and Neck Surgery, PUMCH, CAMS & PUMC, Beijing, 100730 China; 8 Medical Department, PUMCH, CAMS & PUMC, Beijing, 100730 China

**Keywords:** Virtual reality, Anatomy, Medical education

## Abstract

**Background:**

Realistic, portable, and scalable lectures, cadaveric models, 2D atlases and computer simulations are being combined more frequently for teaching anatomy, which result in major increases in user satisfaction. However, although digital simulations may be more portable, interesting, or motivating than traditional teaching tools, whether they are superior in terms of student learning remain unclear. This paper presents a study in which the educational effectiveness of a virtual reality (VR) skull model is compared with that of cadaveric skulls and atlases. The aim of this study was to compare the results of teaching with VR to results of teaching with traditional teaching methods by administering objective questionnaires and perception surveys.

**Methods:**

A mixed-methods study with 73 medical students was conducted with three different groups, namely, the VR group (*N* = 25), cadaver group (*N* = 25) and atlas group (*N* = 23). Anatomical structures were taught through an introductory lecture and model-based learning. All students completed the pre- and post-intervention tests, which comprised a theory test and an identification test. The theory test consisted of 18 multiple-choice questions, and the identification test consisted of 25 fill-in-the-blank questions.

**Results:**

The participants in all three groups had significantly higher total scores on the post-intervention test than on the pre-intervention test; the post-intervention test score in the VR group was not statistically significantly higher than the post-intervention test score of the other groups (VR: 30 [IQR: 22–33.5], cadaver: 26 [IQR: 20–31.5], atlas: 28[IQR: 20–33]; *p* > 0.05). The participants in the VR and cadaver groups provided more positive feedback on their learning models than the atlas group (VR: 26 [IQR: 19–30], cadaver: 25 [IQR: 19.5–29.5], atlas: 12 [IQR: 9–20]; *p* < 0.001).

**Conclusions:**

The skull virtual learning resource (VLR) was equally efficient as the cadaver skull and atlas in teaching anatomy structures. Such a model can aid individuals in understanding complex anatomical structures with a higher level of motivation and tolerable adverse effects.

## Background

Although anatomy serves as the basis for other medical courses for medical students [1], universities have decreased the hours allocated to anatomy education in favour of applied clinical work [2]. Medical students need to supplement their anatomy education with plenty of traditional resources, including cadaveric dissection, preserved specimens, and various 2-dimensional (2D) image representations (e.g. textbook illustrations, atlases, and tomographic scans) [3]. Recent advancements in computer technology have led to many different forms of digital anatomy simulations [4]. Among them, virtual reality (VR) technology is one of the most promising teaching tools in medical education. VR can be used to deliver a highly immersive experience through head-mounted displays (HMDs) and a less immersive experience through a desktop system [5]. A wide range of virtual learning resources (VLRs) have been developed that use 3-dimensional (3D) visualization technologies to supplement and even replace traditional instructional materials such as cadaver dissections [3]. Users can interact with vivid imagery for an active and self-directed learning experience without the limitations of ethical concerns and donation shortages [6, 7] or having to enter an anatomy laboratory [4]. In a few studies, the educational value of VLRs has been compared with that of conventional methods, and the results are generally inconsistent. When used either alone or to complement traditional written and online materials, VLRs showed better or similar effectiveness in terms of enabling students to learn anatomy [2, 8–11]. More importantly, VLRs are rated as more interesting and engaging [8], enjoyable [8, 9, 11], motivating [8, 12–14] and useful for understanding spatial relationships [11, 13, 15] than traditional tools. There is an inherent appeal in these newer and more advanced visualizations in addition to their novelty [4]. However, studies have shown that compared with cadaver dissection and physical modes, VLRs are less effective in improving learning outcomes [16, 17]. The lack of tactile experience is regarded as a disadvantage.

Although current studies suggest that VLRs cannot replace cadaver and physical models, they are perceived as promising supplementary resources in anatomy education. It is therefore important to evaluate the evidence from different aspects. However, current researches are largely focused on the comparison between VLRs and 2D textbooks, online materials or physical models. Petersson et al. [16] and Codd et al. [17] both compared VLRs with cadaver dissection, but neither study used VLRs to deliver a highly immersive experience. Recent studies by Birbara et al. [18] and Shao et al. [10] provide a fully immersive experience, but neither of the research groups compared VLRs with cadaver dissection, and the VLR group used only perception questionnaires. Objective assessments are crucial to evaluating participants’ performance, in which the identification test is considered to be a predictor of improved learning outcomes following 3D learning [19, 20]. Therefore, immersive VLRs and traditional teaching modalities, such as cadaver dissection and 2D atlases, should be further compared, and the different aspects of the assessments should also be considered to evaluate the impact of VLRs on anatomy education.

### Aim and hypothesis

Neurosurgery comprises some of the most challenging surgical procedures, and mastering the intricacies of cranial anatomy is a career-long endeavour for every neurosurgeon [10]. In this study, a coloured and detachable skull VLR was constructed. The aim of this study was to compare the results of the skull VLR with the cadaver skull and a 2D atlas for anatomy education by administering objective questionnaires and perception surveys. Our hypothesis was that the skull VLR and cadaveric skull groups might have similar performance in the objective tests, and that students would show a more positive attitude towards their learning material than the atlas group.

## Materials and methods

### 3D skull model based on VR technology

**Fig. 1:**
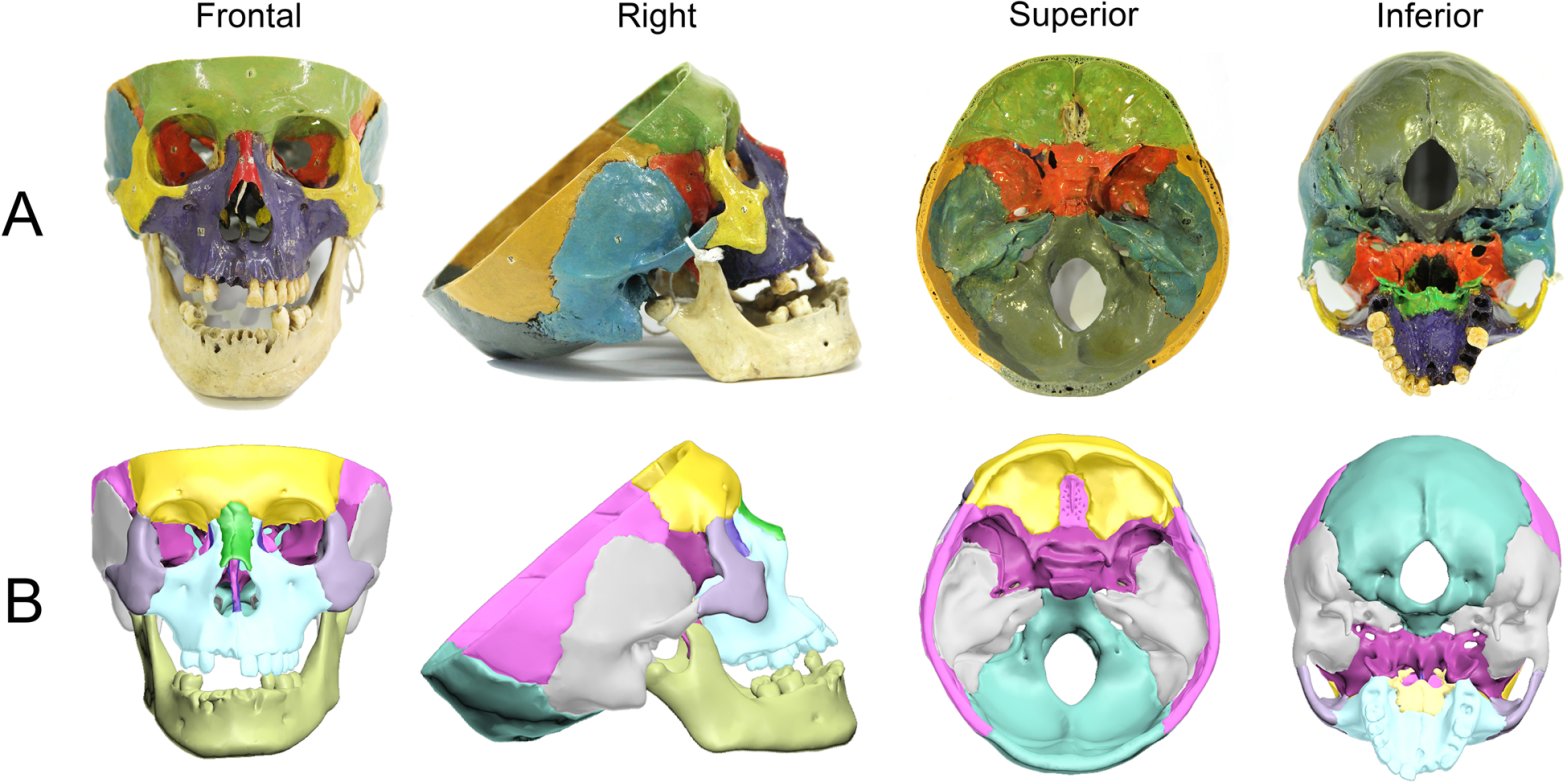
Photos of the cadaveric skull and skull VLR. **a** From left to right, the cadaveric skull is shown in the frontal, right, superior and inferior views. **b** From left to right, the skull VLR is shown in the frontal, right, superior and inferior views

**Fig. 2:**
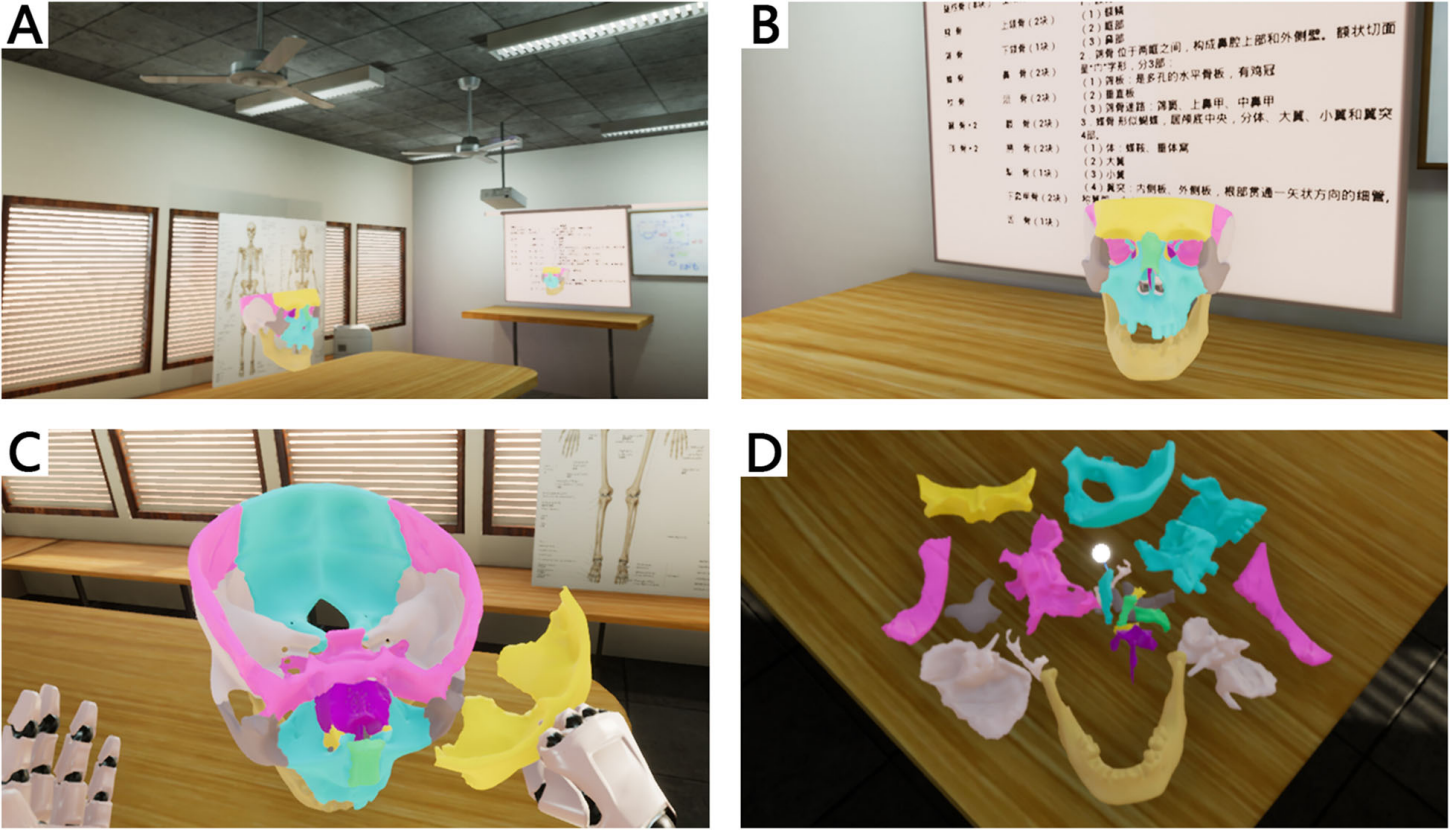
Photos of the simulation classroom and the skull VLR. **a** The entire classroom, in which a skull is placed on a table in the front of the classroom, the other skull is placed on a table in the middle of the classroom, and pictures of the human skeleton are placed in front of the window. **b** The skull VLR and projection screen. **c** The frontal bone separated from the entire skull. **d** All the bones separated, with the bright white ball representing the center of the original skull

The virtual 3D skull model used in this study was constructed from computed tomography (CT) scans of a human skull from the Peking Union Medical College (PUMC) Anatomy Teaching Collection (Fig. 1). The CT scans were imported into Mimics 17.0 (Materialise NV, Leuven, Belgium) and converted into stereolithography (STL) files. The method used to create a 3D model from CT scans was previously published by Shui et al. [21]. Several defective structures (ethmoid plate, crista galli, anterior clinoid process and inferior orbital fissure) on the 3D skull model were modified by using 3D Studio Max 2016 (Autodesk Inc., San Rafael, CA). In addition, each bone was isolated from the entire skull and painted in a different colour (Fig. 2c & d). The model was then imported into the Unreal Engine VR platform (Fig. 2a) through the HTC VIVE software development kit (High Technology Computer Corporation, Taiwan) and Unreal Engine 4.15 (Epic Games Inc., Cary, NC), which is compatible with HTC VIVE CE (High Technology Computer Corporation, Taiwan), a VR HMD with a resolution of 2160 × 1200. Users could rotate and scale the model through handheld controllers. In addition, each cranial bone could be isolated from all other bones, which allows the user to view an individual selected bone and its position in space relative to the other bones. When the isolated structure was placed back in its original position, the model was reset.

### Participants

Seventy-four clinical undergraduates from PUMC who had just finished a 2.5-year pre-medical programme at Tsinghua University were recruited. These students would begin their undergraduate stage of medicine in the subsequent 5.5 years, from the basic study of anatomy to clinical internships. The anatomy course combines regional and systematic anatomy and requires 144 study hours for each student. Every theoretical lecture is followed by a cadaver dissection teaching of equivalent time. There are a theoretical test and an identification test for objective assessment at the end of the course.

The students were randomly divided into three groups, namely, the skull VLR group (VR group, *n* = 25), cadaveric skull group (cadaver group, *n* = 25), and 2D atlas group (atlas group, *n* = 24). Seventy-three participants completed the trial, while one participant in the atlas group dropped out of the study for personal reasons before the pre-intervention test.

### Ethical approval

This study was approved by the Institutional Review Board of the Institute of Peking Union Medical College Hospital (PUMCH) (Project No: ZS-1724).

### Design

**Fig. 3:**
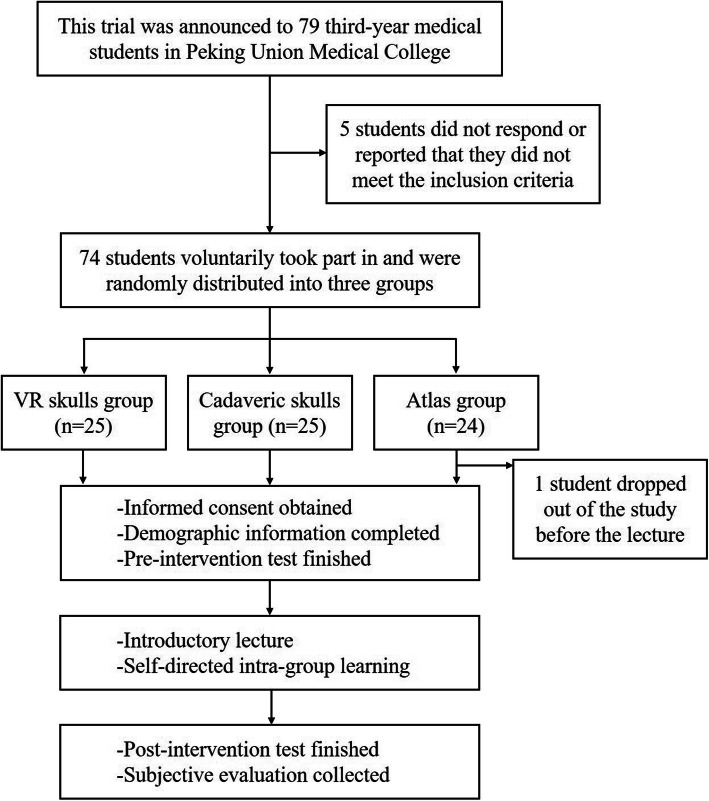
Flowchart of the study design

A flowchart of the study design is displayed in Fig. 3. All participants finished the pre-intervention tests. Then, they attended a 30-min PowerPoint-based introductory lecture on cranial anatomy, which included the characteristics of each cranial bone, feature structures and spatial relationships. The lecture was taught by a teacher from PUMC whom the students had not met before. During the lecture, each participant received a single sheet of paper with the teaching outline, which could also be used for note-taking. Afterwards, the three groups were assigned to three separate rooms for a 30-min self-directed learning session that used skull VLR, cadaveric skulls, and 2D atlases. The students in the VR group received a 2-min instruction about the manipulation of the VR equipment before learning. Study mentors were assigned to each room to prevent intragroup communication and were forbidden to answer questions related to anatomy. The participants took turns so that each participant had 7.5 min to manipulate and observe the model in the first perspective, and they observed the 3D model on the computer screen for the remaining 22.5 min. The participants in the cadaver and atlas groups also had the same amount of time to hold the cadaver skull or atlas, while the other participants could only observe, without manipulation. To compensate for the inability to view the teaching outline on paper in the simulated environment, a projector was used to project the teaching outline on a screen (Fig. 2b). A post-intervention test was conducted immediately after the learning session to evaluate the educational efficacy of each model. Finally, each participant completed a perception survey.

The pre- and post-intervention tests comprised the same set of theory tests and identification tests (Supplementary file 1.1 & 1.2). The theory test consisted of 18 multiple-choice questions that mainly covered basic knowledge on the skull. Each correct answer was awarded 1 point, and the examination lasted 15 min. The identification test consisted of 25 fill-in-the-blank questions on labelled anatomical structures of the skull. All structures were labelled on the cadaveric skulls. The participants had 45 s to observe each structure and write down its name. Each correct answer was awarded 1 point. The content was based on the syllabus from the PUMC anatomy course, and all the test questions are available in Supplementary file 1.

To assess the potential efficacy of the teaching tools, in addition to the objective learning efficiency determined by the test scores, a perception survey was designed (Supplementary file 1.3). The questions were based on those included in several previous studies conducted to evaluate the efficacy of other 3D models [8, 19, 22]. The perception survey used in this study consisted of five parts that addressed the participants’ enjoyment, learning efficiency, attitude, intention to use, and the tool’s authenticity, and a standard five-point Likert scale was used to quantify the responses (1-strongly disagree, 5-strongly agree with the statement).

### Data collection and marking

Demographic information, including each participant’s age, sex, self-reported VR headset experience and video game experience, was collected during the trial. The participants recorded their group and individual identification numbers on the sign-in sheet. The previous grade point average (GPA) of each participant was obtained from the grade counsellor. The demographic and grouping information were hidden from the test mentor, study mentor and study staff until the trial was completed. The study staff scored each answer sheet, and the results were reviewed by the investigators (Zhu J and Cheng C) twice.

By using the Chen et al.’s [19] mean total scores and the variance data of the post-intervention test, power calculations were performed for this study. The calculations revealed that 26 students were required per group (78 students total) to achieve 80% power to detect a 10% change in the post-intervention total scores at an alpha level of 0.05.

### Statistical analysis

The previous GPA, test scores and perception survey scores were expressed as medians (interquartile ranges (IQRs)), and the categorical variables were expressed as numbers (%). The participants’ ages were expressed as means (±SDs). A *p*-value of < 0.05 was considered to indicate statistical significance. Statistical analysis was performed with SPSS 23.0 (IBM Corp, Armonk, NY).

The data distributions were assessed with the Kolmogorov-Smirnov test. The between-group differences in the pre- and post-intervention test scores, changes in the scores, and perception survey scores were assessed with the Kruskal-Wallis H test because they were found to be non-normally distributed. If there was a significant difference with the Kruskal-Wallis H test, the Mann-Whitney U test was employed for pairwise comparisons. The participants’ ages were compared with ANOVA. The categorical variables, except for video game experience, were compared with the chi-square test; video game experience was compared with Fisher’s exact test.

## Results

### Participant demographics

A total of 73 third-year medical students (39 females, 53.42%) were included in the study (Table 1). Most participants were 20 or 21 years old. There were no statistically significant differences across the 3 groups in terms of gender, age, previous GPA in the pre-med programme at Tsinghua University, VR experience or video game experience (all *p* > 0.05).

**Table 1:** Demographic information in the three groups. ^a^Chi-square test. ^b^ANOVA. ^c^Kruskal-Walis H. ^d^Fisher’s Exact test

	VR skulls (*N* = 25)	Cadaveric skulls (*N* = 25)	Atlas (*N* = 23)	*p*-value
Gender [n (%)]				
Male	9 (36%)	13 (52%)	12 (52.17%)	0.425^a^
Female	16 (64%)	12 (48%)	11 (47.83%)	
Age (Median [IQR])	21.22 ± 0.69	21.15 ± 0.54	21.19 ± 0.78	0.948^b^
Previous GPA (Median [IQR])	3.28 [3.14–3.43]	3.30 [3.06–3.47]	3.23 [3.21–3.40]	0.780^c^
VR headset experience [n (%)]	9 (36%)	7 (28%)	7 (30.43%)	0.823^a^
Video game experience [n (%)]				
Always	2 (8%)	0 (0%)	1 (4.35%)	0.696^d^
Occasionally	4 (16%)	4 (16%)	2 (8.70%)	
Rarely	19 (76%)	21 (84%)	20 (86.95%)	

### Comparison of the test scores across groups

The scores for the theory test and identification test were included in the total scores. The maximum scores of the theory test, identification test, and both tests together were 18, 25, and 43 points, respectively. A within-subject analysis showed overall improvement in the test scores from before to after the intervention, and the magnitude of improvement was significantly different across the three groups (*p* < 0.001). Table 2 displays the results of the pre- and post-intervention tests.

**Table 2:** Pre- and post-intervention tests score in the three groups. Full scores of theory test, identification test, and total score were 18, 25, and 43 points, respectively. The median and quartiles of the total scores were not simply equal to the sum of the theory score and the identification score. ^c^Kruskal-Walis H

	VR skulls (*N* = 25)	Cadaveric skulls (*N* = 25)	Atlas (*N* = 23)	*p*-value
Pre-intervention score (Median [IQR])				
Total	9 [6.5–13]	8 [7–11]	10 [7–14]	0.634^c^
Theory test	7 [5–9]	7 [5–9]	7 [6–10]	0.667^c^
Identification test	3 [1.5–4.5]	2 [0.5–3]	2 [1–5]	0.176^c^
Post-intervention score (Median [IQR])				
Total	30 [22–33.5]	26 [20–31.5]	28 [20–33]	0.571^c^
Theory test	15 [12.5–16]	14 [12.5–15.5]	14 [11–16]	0.824^c^
Identification test	15 [10–18]	12 [8–15.5]	13 [8–18]	0.511^c^
Change in score (Median [IQR])				
Total	18 [14.5–21.5]	18 [12.5–21.5]	16 [10–20]	0.317^c^
Theory test	7 [5–9]	7 [4.5–10]	6 [3–8]	0.524^c^
Identification test	12 [8–12]	9 [7.5–13.5[	9 [7–13]	0.278^c^

**Fig. 4:**
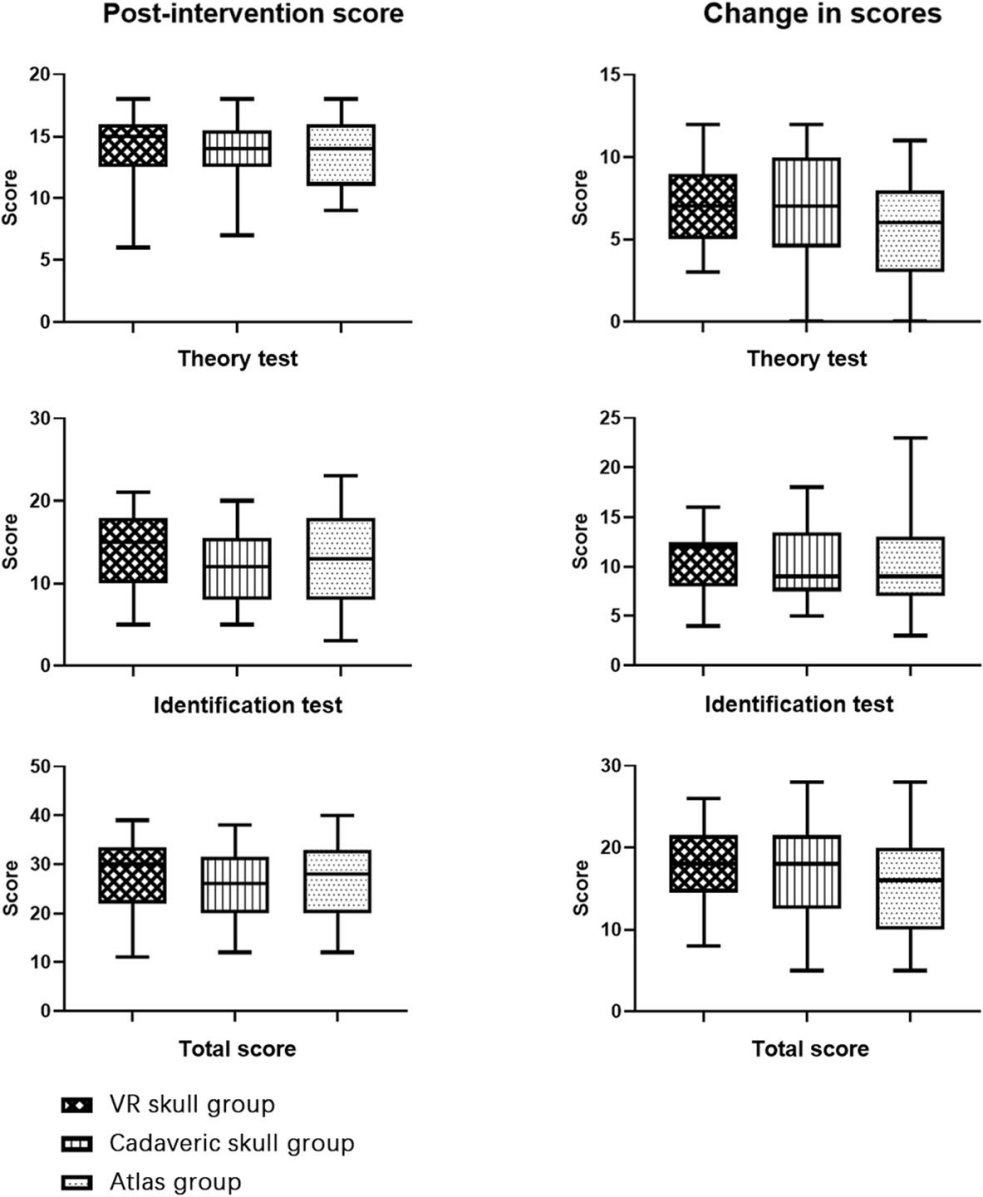
Comparison across the three groups in the post-intervention test scores and changes in scores. There were no statistically significant differences across the three groups in the post-intervention test scores and changes in scores

No statistically significant difference was revealed across the three groups in the pre-intervention tests (*p* > 0.05 for the total score, theory score, and identification score). In terms of the post-intervention test, there were no statistically significant differences across the three groups in either the total score or theory score (all *p* > 0.05). The participants in the VR group performed better in the identification test than the cadaver and atlas groups (Fig. 4), although with no significance (VR: 15 [IQR: 10–18], cadaver: 12 [IQR: 8–15.5], atlas: 13 [IQR: 8–18]; *p* > 0.05).

The differences between the pre- and post-intervention test scores of the individual students were considered by using the change in scores. Kruskal-Wallis H analysis revealed that the changes in the scores were not significantly different across the three groups (*p* > 0.05 for the changes in the total, theory, and identification scores), as shown in Fig. 4.

### Results of the perception survey

Comparisons of the results of the perception survey are shown in Table 3. Overall, the participants in the VR and cadaver groups found their assigned learning models to be more enjoyable (VR: 4 [IQR: 3–5], cadaver: 4 [IQR: 3–5], atlas: 2 [IQR: 1–3]; *p* < 0.001), more interesting (VR: 4 [IQR: 3–5], cadaver: 4 [IQR: 3–4.5], atlas: 2 [IQR: 1–3]; *p* < 0.001), more authentic (VR: 4 [IQR: 3–5], cadaver: 4 [IQR: 3–5], atlas: 2 [IQR: 1–3]; *p* < 0.001), and more efficient for memorization (VR: 3 [IQR: 2–4], cadaver: 3 [IQR: 3–4], atlas: 2 [IQR: 1–4]; *p* < 0.001) and spatial understanding (VR: 4 [IQR: 4–5], cadaver: 4 [IQR: 3–4.5], atlas: 1 [IQR: 1–3]; *p* < 0.001). The VR and cadaver groups reported a higher intention to include the study material for use in standard anatomy education (VR: 3 [IQR: 2–4], cadaver: 4 [IQR: 2.5–4], atlas: 1 [IQR: 1–2]; *p* < 0.001).

**Table 3:** Results of perception survey in the three groups. Full score of perception survey is 35. ^c^Kruskal-Walis H. ^*^p < 0.05

		VR skulls (*N* = 25)	Cadaveric skulls (*N* = 25)	Atlas (*N* = 23)	*p*-value
Enjoyment	Enjoyable	4 [3–5]	4 [3–5]	2 [1–3]	< 0.001^c,*^
	Interest	4 [3–5]	3 [3–4.5]	2 [1–3]	< 0.001^c,*^
Authenticity		3 [2.5–4]	3 [3–4]	2 [1–3]	0.001^c,*^
Learning Efficiency	Memorize	3 [2–4]	3 [3–4]	2 [1–4]	0.029^c,*^
	Spatial	4 [4–5]	4 [3–4.5]	1 [1–3]	< 0.001^c,*^
Attitude		3 [2–3]	3 [2–4]	1 [1–2]	< 0.001^c,*^
Intention to use		3 [2–4]	4 [2.5–4]	1 [1–2]	< 0.001^c,*^
Total		26 [19–30]	25 [19.5–29.5]	12 [9–20]	< 0.001^c,*^

### Discomfort during the learning session

During the learning process, discomfort including headache, blurred vision and nausea were evaluated in the three groups (Supplementary file 2). Although the participants in the VR group exhibited these adverse effects more frequently than in the other 2 groups, no significant difference was found (VR group: 24%, cadaver group: 12%, atlas group: 8.7%, *p* > 0.05). Additionally, in the VR group, the total scores of the post-intervention tests did not vary in the participants with and without discomfort (30 [IQR: 19.25–32.5] vs. 30 [IQR: 22–34], *p* > 0.05).

## Discussion

This is the first study to compare VLR with two different traditional teaching methods in a randomized controlled study design, including both objective assessments and perception surveys, namely, “various question types” in previous research [19]. The results of the objective assessment demonstrated that the skull VLR had the same efficiency as the cadaver skull and atlas in enabling students to learn anatomy, despite the relative simplicity of the model used in this study. The post-intervention identification scores were higher in the VR group, although not significantly, compared with the other two groups. This result was consistent with the advantages of VR in stereoscopic observation and operation, which incorporated the intrinsic spatial relationships of the anatomical sites studied and may thus confer a spatial knowledge advantage [11]. The results of the perception survey in the VR and cadaver groups also showed a more positive attitude towards the learning models than the results of the 2D atlas group, which indicated that the VR and cadaver groups had similar levels of enjoyment, learning efficacy and authenticity. Novel interventions usually spark participants’ curiosity and lead to better results [4], and all participants in the VR group were highly enthusiastic to promote the use of this skull VLR in anatomy education. Similarly, previous studies that have compared a 3D VR model with traditional 2D materials also reported that VR was considered to be a more enjoyable and useful educational tool [8, 11, 23].

Cadavers offer high levels of realism, haptic feedback, and the opportunity to use real instruments and tools, which was found to be superior to atlas models in several previous studies [24–26], particularly in surgery [27]. In our trial, the scores of the cadaver skull group showed no statistically significant differences from the scores of the atlas group. This discrepancy might partially result from structural variations and small damages of the cadaveric skulls and the negative psychological reactions from participants triggered by the cadaveric skulls [28, 29]. In addition, we combined lecture and model learning to simulate the real learning process. The lectures allowed the participants to learn important information for the tests and narrowed the differences across the three groups. Moreover, exposure to the pre-intervention test will affect performance on an identical post-intervention test through familiarity with the questions and may also influence learning during the intervention [30].

3D VLRs, which provide rapid and feedback-based modifications, offer an opportunity for repetitive practice [23]. Another advantage of VLRs is that students can observe and receive instant visual feedback based on predefined practical tasks [31]. Critics have argued that this approach lacks expert guidance during the learning process, which plays an important role in forming the basic framework [32]. In fact, teachers can also assess students’ learning performance and mistakes through digital reports to further improve students’ skills. Moreover, the participants in our trial conducted self-learning in the absence of guidance and gained substantial progress in learning anatomical knowledge, which is consistent with the results in a previous study [33]. It has been suggested that self-learning in private places is a feasible way to implement VR simulation learning without constraints of the place or time provided for learning [33]. In addition, 3D models are likely to enhance rather than replace lecture-based teaching by experts [23]. Individuals can first practice with a VR simulation rather than with cadavers so that they can repeat the procedures and acquire basic skills before using expensive laboratory facilities [33]. Our study incorporated a room-scale HMD unit, which was available for the individuals. With this unit, many more manipulations can be easily achieved, such as rotating to a suitable view, segmenting a single bone and scaling up the model, which makes it a better tool for understanding difficult anatomical structures. This HMD unit provides a completely immersive experience with a high-resolution display, a high refresh rate, and highly precise, low-latency constellation head tracking.

However, adverse effects caused by the highly immersive experience and the device being positioned in front of the eyes were regarded as negative aspects of the VLRs [18]. Adverse effects, including headaches, dizziness, sore eyes, blurred vision and motion sickness, have been reported [2, 34, 35]. A previous study reported a high adverse effect rate in a VR group (headaches 25%; blurred vision 35%) [2], but this rate was lower in our trial (headaches 20%, blurred vision 4%). It can be inferred that the discomfort caused by the virtual environment could be relieved with increased resolution and lower latency. Newer VR designs are being designed to overcome motion sickness and other VR-associated adverse effects. These designs include grounding the user by allowing their eyes to fix on a constant object such as a virtual nose or hand and decoupling the axes of movement from the visual plane [18].

In addition to physical discomfort, the high degree of immersion in a stereoscopic environment and the novel experience of an immersive learning might make the learning process more mentally taxing [11, 15]. Khot et al. suggested that the extraneous load of the virtual delivery modalities might contribute to the worse performance of students who used these modalities than students who used physical models [36]. Birbara et al. also found that students with minimal prior anatomy knowledge in a stereoscopic cohort had a higher cognitive load [18]. With no prior knowledge, the participants in our study might have experienced a greater mental burden, which undermined their learning efficiency in the learning session.

Although commercially available and low-cost HMDs have further enhanced the opportunities for a truly interactive virtual experience [37], the cost of VLR as a supplement should be noted. Each set of VR equipment cost approximately 700 dollars in our study, and it requires related software, computer hardware and technicians for its maintenance [38]. Considering the benefits of improved learning, repeated use, and versatility, VR can reduce the costs of laboratory material, supervisor staff and even simulated patients. In general, the cost-benefit trade-off for these expensive technologies is likely to vary based on institutional goals and resources [39].

### Limitations

Our study had several limitations. First, this is a single-institution study with a small sample size that included only 73 participants, which was close to but smaller than the expected sample size. Further studies with larger sample sizes are needed to examine a broader spectrum of medical personnel including resident physicians, nursing students and related educators. Second, the self-directed learning session was limited to 30 min in our study. The participants in the VR group only received a 2-min instruction for the VR equipment manipulation before the learning session. To reduce the mental burden, prior acquaintance with a virtual environment may be essential in future study designs. Third, as the pre- and post-intervention tests were identical, probability existed that the participants might have purposely focused on the questions in the pre-intervention tests during the 30-min learning session, which may have influenced their performance in the post-intervention tests. Longer learning sessions should be considered in future studies. Finally, this study directly compared VLR with other resources and only combined VLR with traditional lecture. Further studies are needed to identify the optimal combination of VLRs with various teaching methods such as cadaver dissection to fully reflect their effectiveness as a supplementary aid in medical teaching.

## Conclusion

In this study, the skull VLR was equally efficient with cadaver skull and atlas in teaching anatomy structures. Such a model can aid individuals in understanding complex anatomical structures with a high level of motivation and tolerable adverse effects. Advances in 3D digital technology have enabled the development of more sophisticated and realistic VLRs, which provide opportunities for its use in traditional anatomy teaching settings as a powerful supplement. Future generations of medical students may benefit from these technologies at the earliest stages of their learning, from VR anatomy models to patient-specific VR simulations, as needed. Additional studies with larger sample sizes are required not only to evaluate the teaching effectiveness of VLRs in a more comprehensive frame but also to investigate the optimal combinations of VLRs with traditional medical education.

## Supplementary information

**Additional file 1.**

## Data Availability

The datasets used and analysed during the current study are available from the corresponding author on reasonable request.

## Abbreviations

VR: Virtual Reality
VLR: Virtual Learning Resource
2D: Two-dimensional
3D: Three-dimensional
STL: Stereolithography
HMD: Head-mounted Display
GPA: Grade Point Average

## References

[1] Turney BW (2007). Anatomy in a modern medical curriculum. Ann R Coll Surg Engl.

[2] Moro C, Stromberga Z, Raikos A, Stirling A (2017). The effectiveness of virtual and augmented reality in health sciences and medical anatomy. Anat Sci Educ.

[3] Preim B, Saalfeld P (2018). A survey of virtual human anatomy education systems. Comput Graph.

[4] Wainman B, Pukas G, Wolak L, Mohanraj S, Lamb J, Norman GR (2020). The critical role of stereopsis in virtual and mixed reality learning environments. Anat Sci Educ.

[5] Suh A, Prophet J (2018). The state of immersive technology research: a literature analysis. Comput Hum Behav.

[6] Gartner LP (2003). Anatomical sciences in the allopathic medical school curriculum in the United States between 1967-2001. Clin Anat.

[7] Korf HW, Wicht H, Snipes RL, Timmermans JP, Paulsen F, Rune G, Baumgart-Vogt E (2008). The dissection course - necessary and indispensable for teaching anatomy to medical students. Ann Anat.

[8] Stepan K, Zeiger J, Hanchuk S, Del Signore A, Shrivastava R, Govindaraj S, Iloreta A (2017). Immersive virtual reality as a teaching tool for neuroanatomy. Int Forum Allergy Rhinol.

[9] Preece D, Williams SB, Lam R, Weller R (2013). "Let's get physical": advantages of a physical model over 3D computer models and textbooks in learning imaging anatomy. Anat Sci Educ.

[10] Shao X, Yuan Q, Qian D, Ye Z, Chen G, le Zhuang K, Jiang X, Jin Y, Qiang D (2020). Virtual reality technology for teaching neurosurgery of skull base tumor. BMC Med Educ.

[11] Kockro RA, Amaxopoulou C, Killeen T, Wagner W, Reisch R, Schwandt E, Gutenberg A, Giese A, Stofft E, Stadie AT (2015). Stereoscopic neuroanatomy lectures using a three-dimensional virtual reality environment. Ann Anat.

[12] Battulga B, Konishi T, Tamura Y, Moriguchi H (2012). The effectiveness of an interactive 3-dimensional computer graphics model for medical education. Interact J Med Res.

[13] Ferrer-Torregrosa J, Torralba J, Jimenez MA, García S, Barcia JM (2014). ARBOOK: development and assessment of a tool based on augmented reality for anatomy. J Sci Educ Technol.

[14] Ruisoto P, Juanes JA, Contador I, Mayoral P, Prats-Galino A (2012). Experimental evidence for improved neuroimaging interpretation using three-dimensional graphic models. Anat Sci Educ.

[15] Ferrer-Torregrosa J, Jimenez-Rodriguez MA, Torralba-Estelles J, Garzon-Farinos F, Perez-Bermejo M, Fernandez-Ehrling N (2016). Distance learning ects and flipped classroom in the anatomy learning: comparative study of the use of augmented reality, video and notes. BMC Med Educ.

[16] Petersson H, Sinkvist D, Wang C, Smedby O (2009). Web-based interactive 3D visualization as a tool for improved anatomy learning. Anat Sci Educ.

[17] Codd AM, Choudhury B (2011). Virtual reality anatomy: is it comparable with traditional methods in the teaching of human forearm musculoskeletal anatomy?. Anat Sci Educ.

[18] Birbara NS, Sammut C, Pather N (2020). Virtual reality in anatomy: a pilot study evaluating different delivery modalities. Anat Sci Educ.

[19] Chen S, Pan Z, Wu Y, Gu Z, Li M, Liang Z, Zhu H, Yao Y, Shui W, Shen Z, Zhao J, Pan H (2017). The role of three-dimensional printed models of skull in anatomy education: a randomized controlled trail. Sci Rep.

[20] Yammine K, Violato C (2015). A meta-analysis of the educational effectiveness of three-dimensional visualization technologies in teaching anatomy. Anat Sci Educ.

[21] Shui W, Zhou M, Chen S, Pan Z, Deng Q, Yao Y, Pan H, He T, Wang X (2017). The production of digital and printed resources from multiple modalities using visualization and three-dimensional printing techniques. Int J Comput Assist Radiol Surg.

[22] Ryan JR, Chen T, Nakaji P, Frakes DH, Gonzalez LF (2015). Ventriculostomy simulation using patient-specific ventricular anatomy, 3D printing, and hydrogel casting. World Neurosurg.

[23] Lo S, Abaker ASS, Quondamatteo F, Clancy J, Rea P, Marriott M, Chapman P (2020). Use of a virtual 3D anterolateral thigh model in medical education: augmentation and not replacement of traditional teaching?. J Plast Reconstr Aesthet Surg.

[24] Azer SA, Eizenberg N (2007). Do we need dissection in an integrated problem-based learning medical course? Perceptions of first- and second-year students. Surg Radiol Anat.

[25] Kerby J, Shukur Z, Shalhoub J (2011). The relationships between learning outcomes and methods of teaching anatomy as perceived by medical students. Clin Anat.

[26] Patel KM, Moxham BJ (2008). The relationships between learning outcomes and methods of teaching anatomy as perceived by professional anatomists. Clin Anat.

[27] Gold JI, Kim SH, Kant AJ, Joseph MH, Rizzo AS (2006). Effectiveness of virtual reality for pediatric pain distraction during i.v. placement. CyberPsychol Behav.

[28] Snelling J, Sahai A, Ellis H (2003). Attitudes of medical and dental students to dissection. Clin Anat.

[29] Lee YH, Lee YM, Kwon S, Park SH (2011). Reactions of first-year medical students to cadaver dissection and their perception on learning methods in anatomy. Korean J Med Educ.

[30] Cook DA, Beckman TJ (2010). Reflections on experimental research in medical education. Adv Health Sci Educ Theory Pract.

[31] Liu L, Zhou R, Yuan S, Sun Z, Lu X, Li J, Chu F, Walmsley AD, Yan B, Wang L (2020). Simulation training for ceramic crown preparation in the dental setting using a virtual educational system. Eur J Dent Educ.

[32] Silen C, Wirell S, Kvist J, Nylander E, Smedby O (2008). Advanced 3D visualization in student-centred medical education. Med Teach.

[33] Frendo M, Konge L, Caye-Thomasen P, Sorensen MS (2019). Andersen SAW.

[34] Muller-Stich BP, Lob N, Wald D, Bruckner T, Meinzer HP, Kadmon M, Buchler MW, Fischer L (2013). Regular three-dimensional presentations improve in the identification of surgical liver anatomy - a randomized study. BMC Med Educ.

[35] Farra SL, Smith SJ, Ulrich DL (2018). The student experience with varying immersion levels of virtual reality simulation. Nurs Educ Perspect.

[36] Khot Z, Quinlan K, Norman GR, Wainman B (2013). The relative effectiveness of computer-based and traditional resources for education in anatomy. Anat Sci Educ.

[37] Rudran B, Logishetty K (2018). Virtual reality simulation: a paradigm shift for therapy and medical education. Br J Hosp Med (Lond).

[38] Sultan L, Abuznadah W, Al-Jifree H, Khan MA, Alsaywid B, Ashour F (2019). An experimental study on usefulness of virtual reality 360 degrees in undergraduate medical education. Adv Med Educ Pract.

[39] Tomlinson SB, Hendricks BK, Cohen-Gadol A (2019). Immersive three-dimensional modeling and virtual reality for enhanced visualization of operative neurosurgical anatomy. World Neurosurg.

