# Supplementary material for: Can virtual reality improve traditional anatomy education programmes? A mixed-methods study on the use of a 3D skull model
Source: BMC Med Educ. 2020 Oct 31;20:395. doi: 10.1186/s12909-020-02255-6 (PMC7603711; doi:10.1186/s12909-020-02255-6)
Supplement: Supplementary file 1 — Additional file 1. [file 12909_2020_2255_MOESM1_ESM.docx]

**Can virtual reality improve traditional anatomy education programmes? A mixed-methods study on the use of a 3D skull model**

**Shi Chen^1,2^, Jiawei Zhu^3^, Cheng Cheng^3^, Zhouxian Pan^3^, Lingshan Liu^3^, Jianhua Du^3^, Xinhua Shen^7^, Zhen Shen^4^, Huijuan Zhu^1^, Jihai Liu^2,5^, Hua Yang^2,6^, Chao Ma^7^, Hui Pan^1,8^**

^1^Department of Endocrinology, Endocrine Key Laboratory of Ministry of Health, Peking Union Medical College Hospital (PUMCH), Chinese Academe of Medical Sciences & Peking Union Medical College (CAMS & PUMC), Beijing, 100730, China.

^2^National Virtual Simulation Laboratory Education Center of Medical Sciences, PUMCH, CAMS & PUMC, Beijing, 100730, China.

^3^Eight-year Program of Clinical Medicine, PUMCH, CAMS & PUMC, Beijing, 100730, China.

^4^The State Key Laboratory of Management and Control for Complex Systems, Institute of Automation, Chinese Automation, Chinese Academy of Sciences (CASIA), Beijing, 100190, China.

^5^Department of Emergency, PUMCH, CAMS & PUMC, Beijing, 100730, China.

^6^Department of Otolaryngology-Head and Neck Surgery, PUMCH, CAMS & PUMC, Beijing, 100730, China.

^7^Department of Human Anatomy, Histology and Embryology, Institute of Basic Medical Sciences, Neuroscience Center, Chinese Academy of Medical Sciences, School of Basic Medicine, Peking Union Medical College, Beijing, 100005, China.

^8^Medical Department, PUMCH, CAMS & PUMC, Beijing, 100730, China.

Shi Chen, Jiawei Zhu and Cheng Cheng contributed equally to this work. Correspondence to Professor Pan Hui; panhui20111111@163.com

**Supplementary file 1**

**QUESTIONS: Test on cranial anatomy**

1. **Theory test**

Directions: For each question, you must choose the best answer from the four choices marked A, B, C, and D.

| No. | Questions | Answers |
| --- | --- | --- |
| 1 | How many pieces of cranial bones (including facial cranium and cerebral cranium) do human have?  A.18 B.20 C.23 D.28 |  |
| 2 | Which of the following **does not** belong to the cerebral cranium?  A. frontal bone B. zygomatic bone C. parietal bone D. ethmoidal bone |  |
| 3 | Which of the following does not appear in pair?  A. parietal bone B. occipital bone C. temporal bone D. zygomatic bone |  |
| 4 | Lambdoidal suture is made up of and .  A. parietal bone, occipital bone B. parietal bone, frontal bone  C. frontal bone, sphenoid bone D. left parietal bone, right parietal bone |  |
| 5 | Inferior orbital foramen is located on .  A. maxilla B. zygomatic bone C. nasal bone E. mandible |  |
| 6 | Hypophyseal fossa locate on .  A. lamina cribrosa B. orbital plane of frontal bone  C. petrosal part of temporal bone D. body of sphenoid bone |  |
| 7 | Which of the following structures does not participate in the composition of anterior cranial fossa?  A. orbital plane of frontal bone B. lamina cribrosa  C. greater wing of sphenoid bone D. lesser wing of sphenoid bone |  |
| 8 | Which cranial bones participate in the formation of pterion?  A. frontal bone, sphenoid bone, parietal bone and temporal bone  B. frontal bone, occipital bone, parietal bone and temporal bone  C. zygomatic bone, sphenoid bone, parietal bone and temporal bone  D. zygomatic bone, frontal bone, occipital bone and temporal bone |  |
| 9 | Which of the following statement about mandible is **false**？  A. It is composed by ramus of mandible and body of mandible  B. Angulus mandibulae is palpable from the body surface  C. Foramina mandibulae are lateral to rami mandibulae  D. Foramina mentale are anterior lateral to corpus mandibula |  |
| 10 | Which of the following structure is located in the middle cranial fossa?  A. internal acoustic meatus B. trigeminal impression  C. clivus D. petro-occipital fissure |  |
| 11 | Which of the following structure is connected with carotid canal?  A. foramen lacerum B. foramen ovale  C. petro-occipital fissure D. foramen spinosum |  |
| 12 | Which of the following structure **is not** located on the greater wing of the sphenoid bone?  A. foramen spinosum B. foramen ovale  C. foramen rotundum D. optic canal |  |
| 13 | Which of the following structure connect cranial cavity with nasal cavity?  A. optic canal B. foramina ethmoidale  C. foramen rotundum D. foramen spinosum |  |
| 14 | Which of the following structure **is not** located in the posterior fossa?  A. internal acoustic meatus B. foramen lacerum  C. hypophyseal fossa D. jugular foraramen |  |
| 15 | Which of the following structure **does not** belong to the sphenoid body?  A. carotid sulcus B. optic canal  C. tuberculum sellae D. chiasmatic sulcus |  |
| 16 | Which of the following structure **is not** located on the occipital bone?  A. hypoglossal canal B. foramen magnum  C. sulci sigmoideus D. cavernous sinus |  |
| 17 | Which of the following structure is not located on the occipital bone?  A. foramen stylomastoideum B. occipital condyle  C. hypoglossal canal D. culivus |  |
| 18 | Which piece of bone is connected with all cranial bones?  A. ethmoid bone B. sphenoid bone C. temporal bone D. parietal bone |  |

1. **Identification test**

Directions: All structures are on cadaveric skulls.

| No. | Answer | No. | Answer |
| --- | --- | --- | --- |
| 1 |  | 14 |  |
| 2 |  | 15 |  |
| 3 |  | 16 |  |
| 4 |  | 17 |  |
| 5 |  | 18 |  |
| 6 |  | 19 |  |
| 7 |  | 20 |  |
| 8 |  | 21 |  |
| 9 |  | 22 |  |
| 10 |  | 23 |  |
| 11 |  | 24 |  |
| 12 |  | 25 |  |
| 13 |  |  |  |

1. **Subjective evaluation questionnaire**

Direction: 1-strongly disagree, 2-disagree, 3-neutral, 4-agree, 5-strongly agree

| Evaluation to the learning material they used | |
| --- | --- |
| Enjoyment | ①I enjoyed studying with the help of the learning material in our group very much. |
|  | ②It aroused my interests in anatomy study |
| Authenticity | ①It present authentic and integral basal cranial features. |
| Learning efficiency | ①It aided in memorizing. |
|  | ②It aided in spatial comprehension. |
| Attitude | ①It’s the best materials in all three groups. |
| Intention to use | ①It should be promoted to standard basal cranial anatomy education. |

**ANSWERS: Test on cranial anatomy**

1. **Theory test**

| No. | 1) | 2) | 3) | 4) | 5) | 6) | 7) | 8) | 9) |
| --- | --- | --- | --- | --- | --- | --- | --- | --- | --- |
| Answers | C | B | B | A | A | D | C | A | C |
| No. | 10) | 11) | 12) | 13) | 14) | 15) | 16) | 17) | 18) |
| Answers | B | A | D | B | B | B | D | A | B |

1. **Identification test**

| 1 | foramina mandibulae | 14 | olfactory foramina |
| --- | --- | --- | --- |
| 2 | foramen lacerum | 15 | optic canal |
| 3 | lesser wing of sphenoid bone | 16 | inferior nasal concha |
| 4 | petrion | 17 | external occipital protuberance |
| 5 | foramen rotundum | 18 | mastoid process |
| 6 | palate bone | 19 | carotid canal |
| 7 | lacrimal bone | 20 | medial pterygoid plate |
| 8 | hypoglossal canal | 21 | inferior orbital fissure |
| 9 | sigmoid sinus | 22 | occipital condyle |
| 10 | jugular foramen | 23 | zygomatic bone |
| 11 | internal acoustic pore | 24 | mandibular fossa |
| 12 | pituitary fossa | 25 | vomer |
| 13 | petrosal bone |  |  |

**Supplementary file 2**

1. Self-reported discomforts during the learning session

|  | VR Skulls  (N = 25) | Cadaveric skulls  (N = 25) | Atlas  (N = 23) | p-value |
| --- | --- | --- | --- | --- |
| Dizziness | 5 | 3 | 1 | 0.309 |
| Blurred vision | 1 | 0 | 0 | 1.000 |
| Nausea | 2* | 0 | 1 | 0.528 |
| Overall | 6 | 3 | 2 | 0.357 |

***Two participants reported dizziness and nausea at the same time.**

1. Comparison between participants with discomforts and the participants without discomforts in VR group

|  |  | Participants with discomforts  (N=6) | Participants without discomforts  (N=19) | p |
| --- | --- | --- | --- | --- |
| Total score [Median (IQR)] | Post-intervention test | 30 (19.25-32.5) | 30 (22-34) | 0.726 |
|  | Difference between pre- and post-intervention test | 20 (15.5-23) | 17 (14-21) | 0.339 |
| Score of theory test [Median (IQR)] | Post-intervention test | 15 (10.5-16) | 15 (13-16) | 0.747 |
|  | Difference between pre- and post-intervention test | 8.5 (6-9.25) | 7 (5-9) | 0.460 |
| Score of identification test [Median (IQR)] | Post-intervention test | 14 (8.75-17.5) | 15 (10-18) | 0.725 |
|  | Difference between pre- and post-intervention test | 12 (8.75-13.75) | 12 (7-12) | 0.579 |
